# Ten quick tips for classifying unknown bacteriophage

**DOI:** 10.1371/journal.pcbi.1014403

**Published:** 2026-06-23

**Authors:** Fabian T. S. Bastiaanssen, Colin Hill, Andrey N. Shkoporov

**Affiliations:** APC Microbiome Ireland and School of Microbiology, University College Cork, Cork, Ireland; Montreal, CANADA

## Introduction

In microbiome research, bacteriophages (phages) are gaining increased attention for their roles as important ecological actors, as vehicles of horizontal gene transfer, and as “phagebiotics”—potential tools for precision microbiome manipulation: phage cocktails to suppress specific pathogens/pathobionts, “virome transplants” to restore microbiome diversity and functionality, phage vectors for delivery of CRISPR-Cas for microbiome editing. Once referred to as “viral dark matter” [1,2] of the microbiome, complex populations of bacteriophages are becoming easier to sequence and identify thanks to recent advances in high throughput virome sequencing and phage bioinformatics.

Despite this, characterising complex phage populations and individual genomes continues to be a challenge: too many phages, including isolated ones, lack even a partial taxonomic classification, let alone a complete one. If we discussed bacterial taxonomy the way we do viral taxonomy, we might not even know which bacteria are in the same family as *E. coli*.

This is not due to a lack of effort. Unlike the phylogeny of bacteria, there is not a single clade of bacteriophages that we can trace back to the evolutionary origin of viruses. Viruses have multiple origins and appear to be products of convergent evolution [3], and therefore lack universal marker genes, such as 16S rRNA, that can be used to construct a common phylogenetic tree. To make matters worse, not only are there multiple branches to keep organised, but viruses can also exchange genes with evolutionarily unrelated viral species [4] regardless of their genomic organisation [5] or even the nucleic acid type [6]. A single viral genome can therefore encompass multiple and distinct evolutionary histories. With multiple correct answers possible, taxonomy based on this kind of phylogenetic delineation becomes arbitrary. The International Committee on Taxonomy of Viruses (ICTV), the official authority on viral taxonomy, comprises various expert groups that spend their time discussing classification and delimitation criteria. Anyone can propose adjustments or additions to the current viral taxonomy. The appropriate ICTV subcommittees will then review these proposals and vote on whether to approve them.

**Fig 1:**
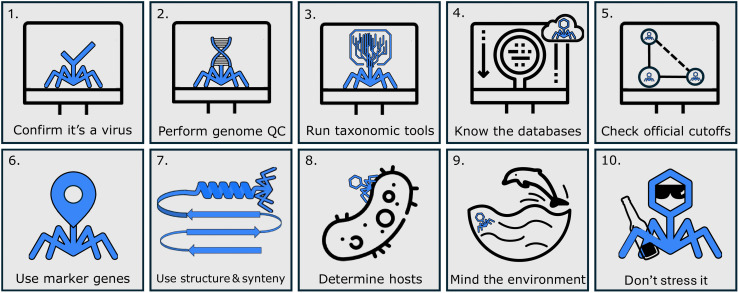
A graphical abstract of the tips.

Given the challenges involved, it might seem tempting not to bother assigning classifications to our genomes and instead wait for others to figure it all out. The benefit of proactive classification should not be overlooked, however; having a sense of taxonomy allows you to link your data to existing literature and databases. While they might be arbitrary, demarcation criteria are, at the end of the day, informed choices that group together similar viruses. If you’re interested in the attributes of the phage, you might find similar traits shared by its related taxa. Alternatively, the taxonomy might inform you of traits you were unaware of. If a related phage is extensively studied for its role in phage therapy, you won’t overlook similar potential in yours. And most importantly, it makes it easier for others to find (and cite) your work in the future. For those with these noble goals in mind, we present a concise and accessible “reader’s digest” in the form of 10 simple tips to guide your path in taxonomically classifying metagenomic and isolated phage genomes (Fig 1). While the principles outlined here apply to phage sequences from any source, we recommend several excellent papers for more specific guidance and further reading [7–9].

## Tip 1: Confirm it is actually a virus

Although this may seem like a straightforward tip, it remains critically important. As demonstrated by the recent publication of the Human Gut Archaeal Virome Database (HGAVD) [10], which elicited a response from researchers highlighting numerous bacterial and archaeal false positives and questioning the reliability of CRISPR-based viral detection methods [11]. This ultimately resulted in a final reply wherein the viral assignments of several genomes were revised a third and final time [12].

While this particular case was exaggerated due to the low number of human gut archaeal viruses in reference databases, thereby ironically highlighting the need for the HGAVD, it still shows the importance of confirming whether a genome is actually viral. The use of proven tools such as Genomad [13], VirSorter2 [14], Jaeger [15] is recommended to determine whether a genome is viral and to separate integrated phage regions from host-derived DNA. After all, finding the right viral classification for a non-virus is a fool’s errand.

## Tip 2: Determine and improve your genome completeness

Due to the small size of viruses and their high level of variability, each uncovered portion of the genome may be of great importance for subsequent analysis steps [16]. It’s therefore in our best interest to recover as much of the original genome as possible. CheckV [17] is currently considered the gold standard tool for assessing the completeness and contamination of viral genomes, however, since CheckV relies on machine learning and is constrained by its training data, manual inspection of low and medium-quality genomes is advisable. For instance, if the assembly graph indicates a closed and therefore probably complete, the low quality might be due to CheckV bias rather than low genome coverage. The simplest way to improve coverage is to increase the number of reads in the assembly, either by increasing the read depth or by pooling multiple sequencing runs. Despite being the most prevalent viruses in the global human gut and present in most individuals, the assembly of the first *Crassvirales* [18–20] genomes were only possible through cross-assembly [21]. This is not to say that higher read counts will necessarily lead to higher genome quality; reads should be randomly subsampled to avoid assembly problems caused by excessive coverage (>50-100x) [22].

To maximise the utility of our sequencing data, we can use post-processing tools such as Phables [23] to resolve the assembly graph and improve the size and quality of assembled contigs. Additionally, tools such as vRhyme [24] or PHAMB [25] can cluster viral contigs that likely originate from the same genome into viral metagenome-assembled genomes (vMAGs).

## Tip 3: Run a taxonomic tool

Taxonomic classification tools such as taxMyPhage [26], Virsorter2 [14], VIRify [27] and vConTACT3 [28] can help narrow down your classification or even complete it. While these tools may not always determine every taxonomic rank, they will, at the very least, give you a push in the right direction. Each method has inherent strengths and weaknesses in classifying different ranks, which, in combination with the limitations of the reference dataset, often result in partial annotations that are biased toward well-studied viral taxa. Combining multiple tools helps compensate for the method-specific differences and can yield a more comprehensive classification [29]. Importantly, the classifications made by machine-learning-based prediction tools require manual verification and should not be considered definitive. If none of the tools can fully classify your genome, congratulations: you may have discovered a novel taxon or an opportunity to establish new parent taxa for the orphan members.

## Tip 4: Curate twice, Blast once

Your genome does not exist in isolation, so don’t classify it as if it does. Referencing existing database entries can be very useful. A natural first stop is the ICTV database, which contains gold-standard, officially recognised viral classifications. Finding an exact or close match can allow you to infer taxonomy from existing classifications, while even distant relatives can provide useful context about shared levels of taxonomy. The number of classified phages is much smaller than that of unclassified phages, and it is quite possible that the ICTV contains no close relatives. Even if your genome has no matches for direct taxonomy inference, larger databases such as NCBI GenBank, IMG/VR [30], UHGV [31], INPHARED [32], PHROGs [33], and VIRE [34] might identify close but not formally recognised relatives. These relatives’ genomes might prove easier to classify in downstream analyses and, through proxy, inform your phage’s classification.

These resources are valuable for identifying sequences, but their taxonomy is unofficial and should be treated cautiously. For example, relying strictly on NCBI’s taxonomic labels can inadvertently exclude close relatives listed as “unclassified viruses” or “unclassified bacterial viruses”, as these are not grouped by phylogenetic or genomic similarity.

The key is balancing relevance with efficiency: include enough sequences to improve your odds of finding a match, but avoid overloading your analysis with unrelated entries, which slow things down.

## Tip 5: Know your cutoffs

The ICTV does not mandate uniform metrics or thresholds. Demarcation criteria are set and enforced for each taxonomic group. Therefore, if you believe your phage belongs to a specific taxon, it is best to review the latest ICTV proposal and determine the current criteria for that taxon. For instance, the ICTV Bacterial and Archaeal Viruses Subcommittee generally recommends approximate cutoffs of ~95% average nucleotide identity (ANI) for species and ~70% for genera [35]. However, this is not universally applicable: for example, members of the order *Crassvirales*, as of MSL #40v2, are classified at the species and genus levels based on the proportion of shared proteins rather than ANI (ICTV Taxonomy proposal 2021.022B.R.Crassvirales). A revision to align *Crassvirales* more closely with the broader viral taxonomy is currently under consideration (ICTV pending proposal 2025.013B.Uc.v4.Crassvirales).

Even when these metrics do not perfectly represent official taxa, having a rough sense of whether your closest matches fall at the species or genus level can substantially improve downstream interpretation. Following the recommended ANI thresholds is particularly helpful during early exploratory analyses, but they must be interpreted with care. Viral genomes vary enormously in size and often share only partially overlapping regions due to extensive horizontal gene transfer. To address this, ANI is sometimes reported alongside coverage, which measures the proportion of the query or reference sequence that aligns with its counterpart, yet this information alone still fails to capture important aspects of relatedness.

For example, take two pairwise comparisons both reporting 95% ANI at 80% coverage. In the first, an 80 kb segment of a 100 kb genome aligns to another 100 kb genome with 95% identity, resulting in roughly 76,000 identical bases and 4,000 mismatches. In the second, a 1 kb genome aligns to 80% of its length (800 bp) in a 100 kb genome at 95% identity, yielding 760 identical bases and 40 mismatches. Although both show 95% ANI and 80% coverage, the actual amount of shared versus divergent sequence differs by nearly two orders of magnitude.

For these reasons, ANI is often more informative when expressed as total ANI (tANI) [9], which accounts for both the coverage of the aligned fraction and alignment length rather than raw identity alone. Tools such as Vclust [36], taxMyPhage [26] and VIRIDIC [35] provide a fast and practical method for calculating ANI, tANI and, related metrics to cluster genomes, providing an initial organisational framework that can be refined later as taxonomy-specific rules are applied.

## Tip 6: Find marker genes for phylogenetics

Although no gene is conserved across all viruses, conserved hallmark genes from a common ancestry are found among members of major viral lineages and will be documented in the dedicated ICTV Reports (https://ictv.global/report). These hallmark genes typically encode proteins involved in replication or virion assembly [37–40]. In tailed bacteriophages (*Caudoviricetes*), examples include the major capsid protein (MCP), portal proteins, and the large terminase subunit (terL), all of which play essential roles in virion assembly or DNA packaging and tend to evolve more slowly than accessory genes. terL is functionally constrained and sufficiently conserved to support alignments across large evolutionary distances, making it especially useful for resolving relationships at the family and order levels. Because phage genomes are highly mosaic, relying on a single marker gene can oversimplify their evolutionary history. A multi-gene approach better captures the composite nature of phage genomes while still focusing on their most evolutionarily stable components.

Phylogenetic trees explicitly model evolutionary relationships and help identify nearest neighbours in a broader context. This is particularly valuable when placing a phage into higher taxonomic ranks, such as families or orders, where genome similarity metrics are less informative. At the same time, it is important to recognise the limitations of phylogenetics: trees based on single or a few genes generally struggle to resolve very recent divergences and are therefore poorly suited to fine-scale distinctions at the species or genus level.

In practice, phylogenetic analysis should be viewed as complementary to genome-wide similarity metrics: trees help establish broad evolutionary placement, while ANI, shared protein content, and other quantitative measures refine relationships at lower taxonomic ranks.

## Tip 7: Favour structure over sequence

**Fig 2:**
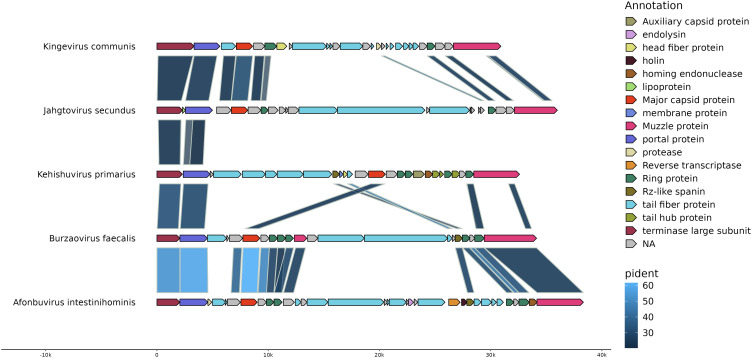
A visualisation of the structural genes within five different members across the families within Crassvirales. Genomes were annotated using Pharokka v1.6 and Prodigal-gv via Pyrodigal, selecting the highest coding density among translation tables 11, 15, and 4. Annotations were further refined using Phold v1 and Phynteny v0.1.12. BLASTp hits (e < 1e-5) between proteins from neighbouring genomes are linked with the brightness of the links scaling with identity.

Just as amino acid sequences are more conserved than nucleotide sequences, patterns of genome organisation preserve evolutionary signal even when individual protein sequences have diverged beyond recognition. For example, *Crassvirales* phage often show conserved blocks of genes involved in replication and virion assembly across genera and families, even where sequence similarity fails (Fig 2). Tools such as vConTACT3 [28] and GRAViTy [41] apply this principle to taxonomically cluster phages with greater precision than just identity alone. Similarly, Phynteny [42] can be used to predict the function of unknown genes by analysing surrounding annotations.

Like genome synteny, protein structures evolve more slowly than their underlying sequences, allowing structural similarity to persist long after detectable sequence identity has been lost to sequence erosion [43]. For instance, many phage integrases display high structural similarity to integrases and recombinases from distantly related organisms, despite sharing less than 25% amino acid identity [44]. Structural phylogeny methods, such as FoldTree [45] leverage this conservation to follow the molecular clock more closely than sequence-only trees and to identify evolutionary signals at deeper levels of the tree.

Until recently, the main challenge for structure-based methods was their high resource and time investments. Although tools like AlphaFold2 [46] and ESMFold [47] are much quicker than experimental methods such as cryo-electron microscopy, they still require significant resources, limiting their use in large-scale comparisons. The computationally costly process of predicting the 3D structure of the protein is mostly wasteful, as the full structure is never used for searches but instead transcribed into a 1D string represented by 20 different characters similar to amino acids, called the 3di alphabet, before any searches and comparisons are possible [48]. ProstT5 [49] tackles this issue by directly predicting 3Di tokens, thereby eliminating the need to first create full 3D models. Benchmark results show that ProstT5 generates 3Di tokens about 50–100 times faster than ESMFold, with only slight decreases in performance [44] and similarity searches using these predicted 3Di tokens can nearly match the sensitivity of structures obtained experimentally and outperform traditional sequence-based methods significantly [49].

## Tip 8: Consider potential hosts

As demonstrated by bacteriophages (i.e., phage that infect bacteria), there is clear value in using host range when discussing and categorising viruses. This principle applies across all levels, assigning a phage to a known host genus (e.g., *Escherichia* phage) generally provides more contextual and comparative utility than an alphanumeric isolate ID. In the case of phage isolate genomes, host assignment is usually clear because the host is inherently linked to isolation and propagation. However, for uncultured metagenomes, identifying hosts is notoriously difficult, relies on prediction tools like iPHoP [50], WIsH [51], and PHIST [52], and should not be considered definitive without experimental validation.

However, while the arms race between host and virus shapes the evolutionary history of both parties, host range does not reliably correspond to monophyletic grouping [53,54]. Due to horizontal gene transfer and specialised mutation strategies enabling rapid host switching, a nonlinear relationship exists between phylogeny and host range. Closely related genotypes may differ in phenotypic host range, while more distantly related viruses may share hosts [55].

Shared or similar hosts may therefore point to evolutionary relatedness, but the absence of a shared host should not be taken as strong evidence against it [29]. That said, host range is most useful when sequence similarity alone cannot resolve taxonomy or when it provides relevant biological context for thresholds based on genomic evidence.

## Tip 9: Consider the environment

Classifications based on environment, such as “marine viruses” or “gut viromes” are often useful and intuitive for biological and clinical purposes. However, as with host range, they should be applied cautiously to taxonomy, as they can produce polyphyletic groups if evolutionary history and horizontal gene transfer are ignored. Furthermore, since viruses from all realms can be detected in a wide range of environments all across the globe, these categories may actually reflect the ecology of their hosts rather than a constraint on the phage itself [56,57]. As a result, related phages may be found in different environments when their hosts overlap, whereas unrelated viruses can co-occur simply because they occupy the same ecological niche.

That said, at lower taxonomic levels, ecology and taxonomy have been shown to intersect. For example, although distributed worldwide at the order level, within the order *Crassvirales* shows a strong correlation between location and phylogeny, with genetically similar members typically found in close geographic proximity [58]. In practice, this means that a marine phage is more likely to resemble other marine inhabitants within its lineage than those found in soil or the human gut.

## Tip 10: Don’t overcomplicate it

At the end of the day, viral taxonomy is a practical tool for conveying shared attributes among related viruses. The choice of ranks and the boundaries between them are human-made constructs for describing the spectrum of viral diversity, which inevitably entails sacrificing some information for utility. Classifications that encompass insights into the biology or ecology of a monophyletic clade are much better than classifications based on thresholds alone.

We should avoid delaying a serviceable classification by attempting to force a “perfect” and exhaustive classification scheme. While additional ranks provide additional information, under the ICTV, the only mandatory ranks are species and genus, which provide sufficient structure for most purposes. If your analysis reveals interesting features of a species, its ecology, or functional repertoire, a partial but actionable classification for its closest relatives is more useful now than a precise higher-rank placement in the distant future. Further refinement of the classification is not only possible, but inevitable as new species are discovered. Taxonomy is the means to an end, not the end by all means.

## Conclusion

Effective viral taxonomy is cumulative, relying on preexisting knowledge and on the rigour with which it is applied to new data. The more rigorously and consistently we classify newly discovered phages, the easier it becomes to place future isolates within a coherent and informative framework. Each well-justified classification strengthens the reference landscape on which all subsequent taxonomic decisions depend.

At the same time, taxonomy is vulnerable to compounding errors, as incorrect or overly confident annotations can spread through databases, bias comparative analyses, and obscure true evolutionary relationships. Because viruses lack fixed, objective thresholds for delimiting taxa, poorly justified classifications can have long-lasting adverse effects on future research. One way to mitigate this risk is to employ multiple methods and compare their classifications. Agreement between methods increases confidence in taxonomic assignments, whereas discrepancies can reveal methodological limitations or biologically interesting edge cases.

When groupings make biological and evolutionary sense, they should be adopted; when they do not, the rules and criteria should be revisited and refined as more data become available. Used thoughtfully, taxonomy is a powerful tool for understanding viruses. Used rigidly, it risks becoming an obstacle rather than an aid.

Lastly, when in doubt, you can always reach out to the ICTV. ICTV members are generally approachable, supportive, and willing to assist researchers by answering questions or directing them toward the most appropriate resources and recommendations.
